# Hemolysin Co-regulated Family Proteins Hcp1 and Hcp2 Contribute to *Edwardsiella ictaluri* Pathogenesis

**DOI:** 10.3389/fvets.2021.681609

**Published:** 2021-06-02

**Authors:** Safak Kalindamar, Hossam Abdelhamed, Adef O. Kordon, Lesya M. Pinchuk, Attila Karsi

**Affiliations:** ^1^Department of Molecular Biology and Genetics, Faculty of Arts and Sciences, Ordu University, Ordu, Turkey; ^2^Department of Comparative Biomedical Sciences, College of Veterinary Medicine, Mississippi State University, Mississippi State, MS, United States

**Keywords:** T6SS, EvpC, Hcp, macrophages, CCO cells, virulence, *Ictalurus punctatus*

## Abstract

*Edwardsiella ictaluri* is a Gram-negative facultative intracellular pathogen causing enteric septicemia of catfish (ESC), a devastating disease resulting in significant economic losses in the U.S. catfish industry. Bacterial secretion systems are involved in many bacteria's virulence, and Type VI Secretion System (T6SS) is a critical apparatus utilized by several pathogenic Gram-negative bacteria. *E. ictaluri* strain 93–146 genome has a complete T6SS operon with 16 genes, but the roles of these genes are still not explored. In this research, we aimed to understand the roles of two hemolysin co-regulated family proteins, Hcp1 (EvpC) and Hcp2. To achieve this goal, single and double *E. ictaluri* mutants (*Ei*Δ*evpC, Ei*Δ*hcp2*, and *Ei*Δ*evpC*Δ*hcp2*) were generated and characterized. Catfish peritoneal macrophages were able to kill *Ei*Δ*hcp2* better than *Ei*Δ*evpC, Ei*Δ*evpC*Δ*hcp2*, and *E. ictaluri* wild-type (*Ei*WT). The attachment of *Ei*Δ*hcp2* and *Ei*Δ*evpC*Δ*hcp2* to ovary cells significantly decreased compared to *Ei*WT whereas the cell invasion rates of these mutants were the same as that of *Ei*WT. Mutants exposed to normal catfish serum *in vitro* showed serum resistance. The fish challenges demonstrated that *Ei*Δ*evpC* and *Ei*Δ*evpC*Δ*hcp2* were attenuated completely and provided excellent protection against *Ei*WT infection in catfish fingerlings. Interestingly, *Ei*Δ*hcp2* caused higher mortality than that of *Ei*WT in catfish fingerlings, and severe clinical signs were observed. Although fry were more susceptible to vaccination with *Ei*Δ*evpC* and *Ei*Δ*evpC*Δ*hcp2*, their attenuation and protection were significantly higher compared to *Ei*WT and sham groups, respectively. Taken together, our data indicated that *evpC* (*hcp1*) is involved in *E. ictaluri* virulence in catfish while *hcp2* is involved in adhesion to epithelial cells and survival inside catfish macrophages.

## Introduction

*Edwardsiella ictaluri* (*E. ictaluri*) is the causative agent of enteric septicemia of catfish (ESC) (1). Although *E. ictaluri* is well-adapted to catfish, it can also infect other freshwater fish species (2–4). At the early stages of host invasion, *E. ictaluri* encounters the host immune system (5, 6). However, *E. ictaluri* is capable of surviving and replicating inside catfish professional phagocytic cells, macrophages, and neutrophils (7). To replicate successfully inside the host cells, *E. ictaluri* must resist and overcome the bacterial killing mechanisms present in the host macrophages and neutrophils, such as oxidative and nitrosative stress (8–10).

The survival of *E. ictaluri* highly depends on its resistance to host stress factors and modulating the host environment. *Edwardsiella ictaluri* encodes urease that is activated in acidic phagosomes of macrophage to cope with low pH (11, 12). Low pH and low phosphate concentration inside the phagosome can trigger the expression level of genes in both Type III and Type VI secretion systems (T3SS and T6SS), which assists *E. ictaluri* survival inside the host immune cells (13). It was shown that the effector proteins secreted via T3SS had an important role in virulence of *E. ictaluri*, and mutation of these genes caused decreased intracellular replication inside catfish head kidney-derived macrophages (14, 15).

Hemolysin co-regulated family proteins (Hcp) are involved in bacteria-host interaction. Particularly, they are involved in adhesion and invasion, intracellular survival of bacteria, bacterial cytotoxicity, and virulence (16). In *E. ictaluri*, T6SS proteins, Eip19 (*evpE*), Eip18 (*evpC*), Eip55 (*evpB*), Eip20 (*evpA*), have been first identified during the catfish host-pathogen interaction (17). The secretion of *evpC* is transcriptionally controlled by two-component system regulatory protein *esrC* in low-pH and phosphate conditions in *E. ictaluri* (13). Fish pathogen *E. piscicida* also possesses T6SS, which is required for virulence (18). In *E. tarda, evpC* plays a dual role as a chaperone and T6SS-dependent secreted protein (19). *evpC* belongs to Hcp family proteins and can bind to T6SS-dependent effector proteins in bacterial cytoplasm and guide effector proteins through the T6SS needle (20). Due to their role as a chaperone protein, *evpC* interacts with the T6SS-dependent effector proteins such as *evpP* in *E. tarda* (21). A recent study showed that *evpP* effector protein secreted via *evpC* could target the macrophages' inflammasome activation (22).

*E. ictaluri* genome has a complete T6SS operon with *evpC* (*hcp1*) while *hcp2* is located outside of the T6SS operon. In this study, we evaluated the role of *hcp* genes in *E. ictaluri*-catfish interaction. Our study revealed roles of *evpC* and *hcp2* in adhesion and invasion of catfish epithelial cells, survival and replication inside catfish peritoneal macrophages, adaptation to the stress factors, and virulence and efficacy in catfish.

## Materials and Methods

### Bacteria, Plasmids, and Media

Bacterial strains and plasmids used in this work were listed in Table 1. *Edwardsiella ictaluri* strain 93–146 (*Ei*WT) and isogenic *hcp* mutants were grown at 30°C in Brain Heart Infusion (BHI) broth or agar. *Escherichia coli* (*E. coli*) CC118λ*pir*, BW19851 (Δ*uidA*3::*pir*), and DH5α strains were cultured on Luria–Bertani (LB) agar or broth and incubated at 37°C. Antibiotics were added to the culture medium at the following concentrations: gentamicin (10 μg/ml), ampicillin (100 μg/ml), and colistin (12.5 μg/ml).

**Table 1:** Bacterial strains and plasmids.

**Strain or plasmid**	**Description**	**References**
***Edwardsiella ictaluri***		
93-146	Wild-type; pEI1; pEI2; Col^r^	(23)
*Ei*Δ*evpC*	93–146 derivative; pEI1; pEI2; Col^r^, Δ*evpC*	This study
*Ei*Δ*hcp2*	93–146 derivative; pEI1; pEI2; Col^r^, Δ*hcp2*	This study
*Ei*Δ*evpC*Δ*hcp2*	93–146 derivative; pEI1; pEI2; Col^r^, Δ*evpC*Δ*hcp2*	This study
***Escherichia coli***		
CC118λ*pir*	Δ*(ara-leu); araD;* Δ*lacX74; galE; galK; phoA20;* *thi-1; rpsE; rpoB; argE(Am); recAl; λpirR6K*	(24)
BW19851λ*pir*	*RP4-2 (Km::Tn7, Tc::Mu-1), DuidA3::pir+, recA1, endA1, thi-1, hsdR17, creC510*	(25)
DH5α	dlacZ Delta M15 Delta(lacZYA-argF) U169 recA1 endA1 hsdR17(rK-mK+) supE44 thi-1 gyrA96 relA1 (2)	(26)
**Plasmids**		
pMEG375	8,142 bp, Amp^r^, Cm^r^, lacZ, R6K ori, mob incP, sacR sacB	(27)
pEiΔ*evpC*	9,939 bp, pMEG-375, Δ*evpC*	This study
pEiΔ*hcp2*	9,939 bp, pMEG-375, Δ*hcp2*	This study
pEiΔ*evpC*Δ*hcp2*	9,939 bp, pMEG-375, Δ*evpC*Δ*hcp2*	This study
pAK*gfplux1*	5,681 bp, PstI, EcoRI, HpaI, AseI, BstBI	(28)

### In-frame Deletion of *evpC* and *hcp2*

The nucleotide sequences of *evpC* (NT01EI_RS11900) and *hcp2* (NT01EI_RS14960) were obtained from the *E. ictaluri* 93–146 genome (GenBank accession: 95 CP001600) (29). The overlap extension PCR method was used to generate *evpC* and *hcp2* in-frame deletion fragments. Briefly, external and internal primers were designed to amplify the regions for upstream and downstream of each gene (Table 2). Two amplified fragments were combined through splicing by overlap extension (SOEing) (30). The overlap PCR product and the pMEG375 suicide plasmid were digested with the same restriction enzymes, and the mutated insert was ligated into the pMEG375. After electroporation and selection of the correct plasmid in CC118, the plasmid was transferred to *E. coli* BW19851 by electroporation, which was then used to transfer the plasmid into *E. ictaluri s*train 93–146 by conjugation. Two-step selection was used to obtain in-frame deletion mutants. At the first step, ampicillin-resistant *E. ictaluri* colonies were inoculated into BHI broth containing ampicillin and colistin. In the second step, positive colonies were streaked on the BHI agar containing colistin only. These colonies were re-streaked on the BHI agar with 5% sucrose, 0.35% D-mannitol, and colistin. Ampicillin-sensitive colonies with the mutant band were in-frame deletion colonies. The deletion of each gene was confirmed by PCR and sequencing. For the construction of double mutant, *E. coli* BW19851 carrying pMEG375 with overlap *hcp2* and *Ei*Δ*evpC* were conjugated, and two-step selection yielded *Ei*Δ*evpC*Δ*hcp2*. PCR and sequencing confirmed the deletion of *hcp2* in the double mutant.

**Table 2:** Primers used for in-frame deletion.

**Primers**	**Sequence (5′ to 3′)^**a**^**	**RE^**b**^**
*Ei*Δ*evpC*EF01	cccc**tctaga**ATCGGGGATTATGAGTTCAGC	*Xba*I
*Ei*Δ*evpC*IR01	ggaacggtacagggtgacatatAGCGGACCTCTCTTGTGAC	
*Ei*Δ*evpC*IF01	ATATGTCACCCTGTACCGTTCC	
*Ei*Δ*evpC*ER01	cccc**ggatcc**CAGTCCCACCATGATAAAGC	*Bam*HI
*Ei*Δ*hcp2*EF01	ccc**tctaga**ACAGGCCAACAAAATTCTCGC	*Xba*I
*Ei*Δ*hcp2*IR01	gtcagagggggtatttgcttcGACTACCGGAGAGCCATTCTC	
*Ei*Δ*hcp2*IF01	GAAGCAAATACCCCCTCTGAC	
*Ei*Δ*hcp2*ER01	ccc**gagctc**GTGGTGTACCGAGAACCACTG	*Not*I

a *Bold letters show restriction enzyme recognition sequences added to primers. Underlined letters indicate reverse complemented primer sequences*.

b *Restriction enzyme*.

### Hemolysis Assay

*Ei*Δ*evpC, Ei*Δ*hcp2, Ei*Δ*evpC*Δ*hcp2*, and *Ei*WT were streaked on sheep blood agar plates (Fisher Scientific), which were incubated at 30°C for 48 h. Hemolytic activity of the mutants was visualized using a Stuart Colony Counter with sub-stage illumination (Cole-Parmer).

### Construction of Bioluminescent Strains

pAK*gfplux*1 was used to construct bioluminescent *Ei*Δ*evpC, Ei*Δ*hcp2, and Ei*Δ*evpC*Δ*hcp2* strains, as described previously (28). Briefly, *E. coli* SM10λ*pir* carrying pAK*gfplux*1 and mutants were grown overnight and mixed at the ratio of 1:2 (donor: recipient). Mixture pellet was spotted on 0.45 μM filter paper placed on BHI agar and grown at 30°C for 24 h. Filter paper containing a mixture of bacteria was washed with BHI broth containing ampicillin and colistin, and serial dilutions were spread on selective BHI agar containing ampicillin and colistin. Ampicillin resistant mutant colonies carrying pAK*gfplux*1 appeared on the selective plates after 30°C for 24–48 h.

### Serum Treatment

Bioluminescent *Ei*Δ*evpC, Ei*Δ*hcp2*, and *Ei*Δ*evpC*Δ*hcp2* strains were exposed to catfish normal serum. Bioluminescent *Ei*WT (positive control) and *E. coli* DH5α (negative control) were also included in each experiment. Catfish serum was collected as previously described (28). Then, 195 μl catfish serum was added to each well of a 96 black well-plate (Corning Costar). Next, 5 μl of the overnight bacterial culture [optical density at 600 nm (OD_600_) = 1.0] was mixed with the serum and inoculated for 4 h at 30°C. The experiment included four replicates, and bioluminescence was measured by using SpectraMax M5 Multi-Mode Microplate Reader (Molecular Devices). Bioluminescent images were taken using an IVIS Lumina XRMS In Vivo Imaging System Series III (PerkinElmer).

### Bioluminescent Imaging

Sixteen specific-pathogen-free (SPF) catfish fingerlings (12.72 ± 1.00 cm, 24.95 ± 5.47 g) were obtained from the CVM hatchery and stocked into four tanks (4 fish/tank). Three tanks were assigned to *Ei*Δ*evpC, Ei*Δ*hcp2*, and *Ei*Δ*evpC*Δ*hcp2* (treatments), and one tank for *Ei*WT (positive control). After 1 week of acclimatization, the water level was reduced to 10 L, and 100 ml bacterial culture was added to each tank (final dose of 5 × 10^7^ colony forming units, CFU, per ml of water). Following 1 h incubation, water flow was restored in each tank. Fish were anesthetized with 100 mg/L MS222, and bioluminescence emitted from the fish body was collected for one min by using IVIS Lumina XRMS In Vivo Imaging System Series III (PerkinElmer). Following bioluminescent imaging, fish were transferred to buckets with aerated water for recovery. Bioluminescent imaging was conducted at 0, 6, 12, and 24 h post-infection, and subsequent daily intervals until 14 days.

### Bacterial Killing Assay

The bacterial killing assay was performed as previously described (12, 31, 32). Briefly, peritoneal macrophages were collected from a year-old channel catfish (250–300 g) injected with 1 ml squalene (Sigma). Following 4-day post-injection, peritoneal macrophages were harvested from five catfish by injecting 10 ml cold phosphate-buffered saline (1X, PBS) to the peritoneal cavity of catfish. Harvested cells were pooled and washed with PBS three times. The cells were resuspended in channel catfish macrophage medium (CCMM) including RPMI (RPMI 1640 sans phenol red & L-glutamine, Lonza) containing 1× glutamine substitute (GlutaMAX –I CTS, Invitrogen), 15 mM HEPES buffer (Invitrogen), in 0.18% sodium bicarbonate solution (Invitrogen), 0.05 mM 2-beta-mercaptoethanol (Sigma), and 5% heat-inactivated (HI) pooled channel catfish serum. Next, peritoneal macrophages (5 × 10^5^ cells) were transferred into a 96-well plate (Evergreen Scientific), and bioluminescent *E. ictaluri* strains were added at a 1:1 ratio and mixed gently by pipetting up and down. The final volume of the cell-bacteria mixture was 200 μl in each well, and the plate included four replicate wells for each treatment and negative control (cell only). The plate was centrifuged at 1,500 rpm for 5 min at 24°C to compact the cells and bacteria at the bottom. The plate was then incubated for 1 h at 30°C to allow the invasion of catfish peritoneal macrophages by bioluminescent mutants and *Ei*WT. Following the first incubation, the cell-bacteria mixture was centrifuged at 2,000 rpm for 7 min, and the media was removed. After this, CCMM containing 100 μg/ml gentamicin were added, and cells were incubated an additional 1 h at 30°C to kill non-phagocyted *E. ictaluri*. At the end of incubation, each well was washed three times with PBS, and peritoneal macrophages were suspended in CCMM with 10 μg/ml gentamicin. Finally, cells were transferred to black 96-well-plates (Fisher Scientific), and the plate was placed in Cytation 5 (BioTek) where the cells were incubated for 48 h under 5% CO_2_ at 30°C. Bioluminescence was captured every hour, and data were analyzed to determine the number of survived bioluminescent bacteria in catfish peritoneal macrophages.

### Attachment and Invasion Assays

Attachment and invasion assays were performed by using channel catfish ovary (CCO) cell line, as described previously (33). Briefly, CCO cells were resuspended in DMEM medium (Sigma) supplemented with 10% fetal bovine serum and 4 mM L-glutamine at a final concentration of 1 x 10^7^ cells ml^−1^. Bioluminescent mutants and *Ei*WT were mixed with CCO cells at a 1:1 ratio and placed in a 24-well-plate. The cell-bacteria mixture's final volume was 1 ml in each well, and the plate included four replicate wells for each treatment and negative control (no bacteria). The plate was incubated 1 h at 28°C for the attachment of mutants and *Ei*WT to CCO. After that, the cell suspensions were incubated in DMEM containing 100 μg/ml gentamicin for 1 h to kill the external bacteria. The plate was washed with PBS three times, and the invasion of *E. ictaluri* strains was determined by imaging IVIS Lumina XRMS In Vivo Imaging System Series III (PerkinElmer).

### Stress Assays

The mutants' survival in oxidative stress in hydrogen peroxide (H_2_O_2_) (Sigma) and nitrosative stress in sodium nitroprusside (SNP) (Sigma) were tested in BHI (rich medium) and low phosphate minimal medium at pH 5.5 (MM19-P) (34). Bacteria were grown overnight, and OD_600_ adjusted to 0.5 for each culture. Five microliter of bacteria from each strain were inoculated into 195 μl of BHI and MM19-P broth containing 0.75 mM H_2_O_2_ (diluted from 30% stock solution) and 5 mM SNP. Each 96-well-black plate included three replicates for each mutant and *Ei*WT as a positive control. The mean photon counts for each stress treatment were measured after 4, 8, 12, and 24 h incubation at 30°C by using IVIS Lumina XRMS In Vivo Imaging System Series III (PerkinElmer).

### Virulence and Efficacy of Mutants in Catfish Fingerlings and Fry

Vaccination and efficacy were performed as previously described (35). Briefly, specific-pathogen-free (SPF) channel catfish fingerlings and fry were obtained from the MSU-CVM Hatchery. Catfish fingerlings (10.46 ± 0.86 cm, 14.03 ± 3.57 g) were stocked into 15 tanks at a rate of 25 fish/tank. Catfish fry were stocked in 12 tanks at a rate of 50 fish/tank. Fish were acclimated at 26–28°C for 1 week and fed twice a day. Chlorine, dissolved oxygen, and temperature were monitored daily. Treatments were randomly assigned to *Ei*Δ*evpC, Ei*Δ*hcp2, Ei*Δ*evpC*Δ*hcp2* (vaccination), *Ei*WT (positive control), and BHI (sham) groups. Each treatment had three replicates. Immersion vaccination was applied by lowering the water level in each tank to 10-L, and by adding 100 ml of bacterial culture (final dose of 2.4 × 10^7^ CFU/ml water). After 1 h, water flow (1 liter/min) was restored to each tank. Mortalities were recorded daily for 21 days, and the percent mortalities were calculated for each group. To assess the protective capabilities of mutants, all fish that survived the *Ei*Δ*evpC, Ei*Δ*hcp2*, and *Ei*Δ*evpC*Δ*hcp2* vaccination were re-challenged with *Ei*WT (2.8 × 10^7^ CFU/ml) 21 days post-vaccination as described above. Fish mortalities were recorded daily, and the experiment was terminated when no fish mortalities were observed for three consecutive days.

### Statistical Analysis

The significance of the differences between treatment means was established by one-way ANOVA and two-way ANOVA procedures with Tukey's test in SAS for Windows 9.4 (SAS Institute, Inc., Cary, NC). The level of significance for all tests was set at *p* < 0.05.

## Results

### Hemolytic Activity of the Mutants

**Figure 1:**
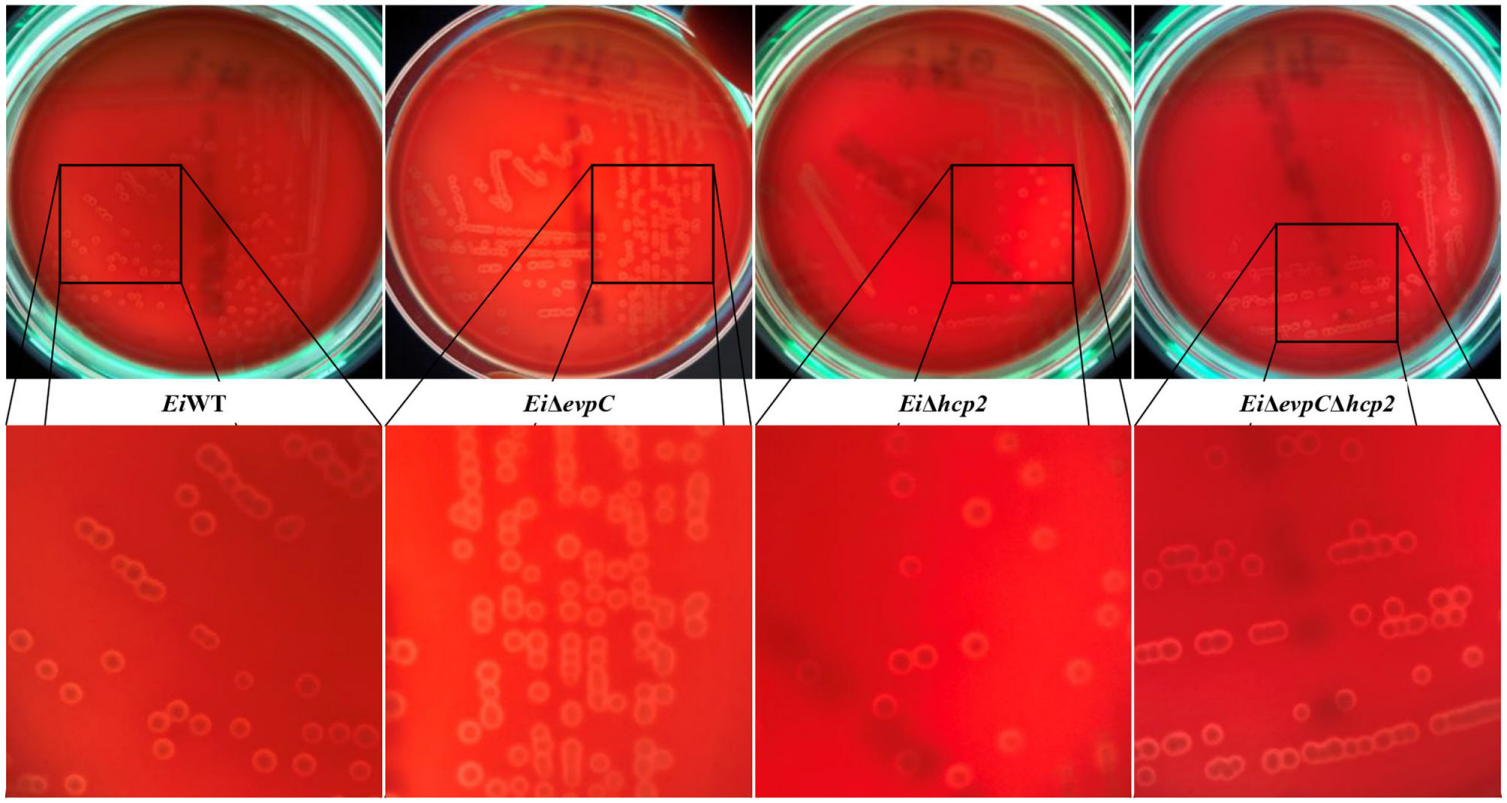
Hemolytic activities of *Ei*Δ*evpC, Ei*Δ*hcp2, Ei*Δ*evpC*Δ*hcp2*, and *Ei*WT on blood agar plates.

A beta-hemolysis with a narrow clear hemolytic zone around the colonies was observed, and hemolytic activity of *Ei*Δ*evpC, Ei*Δ*hcp2*, and *Ei*Δ*evpC*Δ*hcp2* was similar to *Ei*WT (Figure 1).

### Survival of the Mutants Under Complement Stress

**Figure 2:**
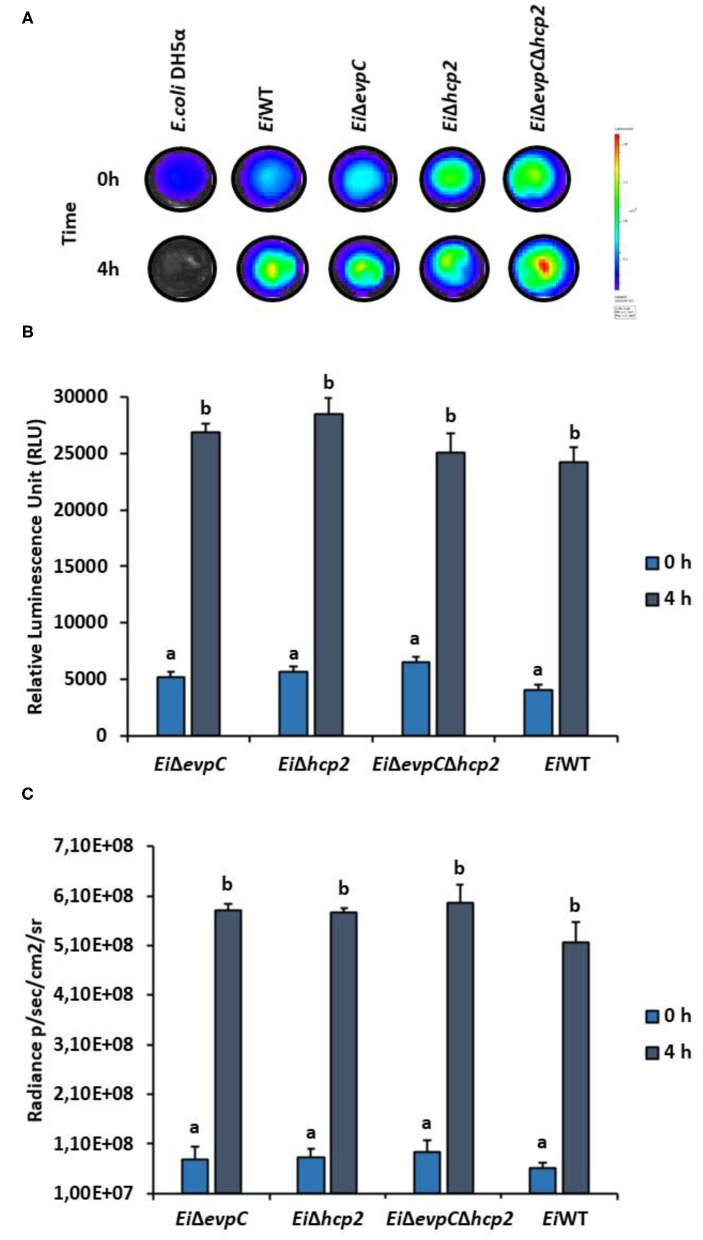
Treatment of *Ei*Δ*evpC, Ei*Δ*hcp2, Ei*Δ*evpC*Δ*hcp2*, and *Ei*WT with normal catfish serum. **(A)** Bioluminescence of mutants, *Ei*WT, and *E. coli* DH5α (negative control) treated with normal catfish serum at 0 and 4 h. Total photon emissions were collected by IVIS Lumina XRMS In Vivo Imaging System Series III at an exposure time of 1 min. **(B)** The bar graph indicates the relative luminescence unit (RLU) obtained by Cytation 5 from the mutants and *Ei*WT treated with the catfish normal serum. **(C)** Total photon counts obtained from bioluminescent image in **(A)**. Data represent the mean of each treatment ± SD. Letters (a and b) show significant differences between treatments at each time points (0 and 4 h) (*p* < 0.05).

Channel catfish serum was used to evaluate the survival of mutants under complement stress. *Ei*Δ*evpC, Ei*Δ*hcp2*, and *Ei*Δ*evpC*Δ*hcp2* were able to survive after 4 h of incubation in catfish serum (Figure 2A), and no significant differences have been detected between the mutants and *Ei*WT (Figure 2B). However, significant differences in the intensity of bacterial bioluminescence were found between 0 and 4 h (*p* < 0.05; Figure 2C). These results indicate that *Ei*WT and mutant strains were robust to complement killing and able to replicate in catfish serum.

### Persistence of the Mutants in Catfish Fingerlings

**Figure 3:**
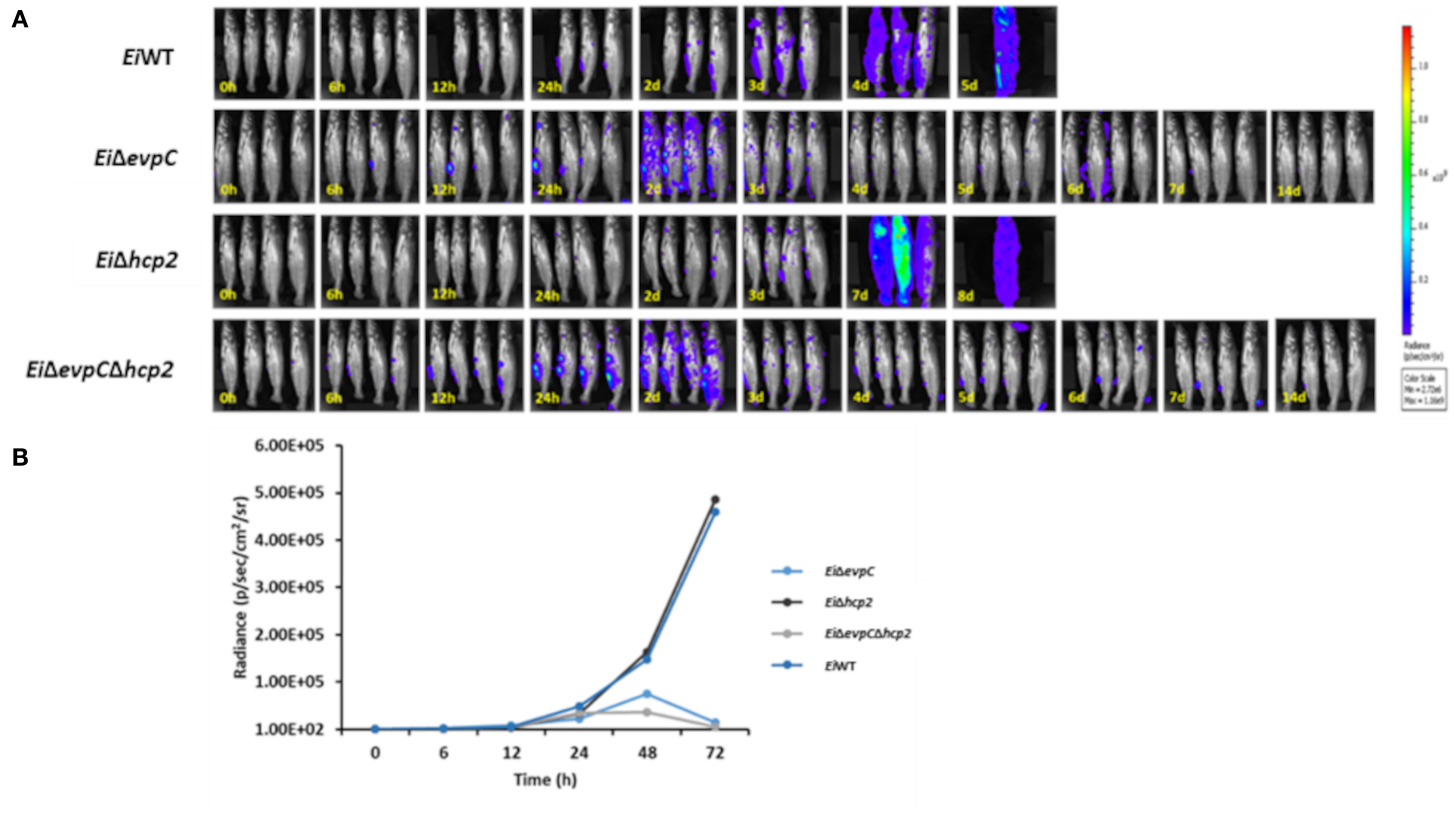
Bioluminescent imaging of live catfish fingerlings challenged with *Ei*Δ*evpC, Ei*Δ*hcp2, Ei*Δ*evpC*Δ*hcp2*, and *Ei*WT. **(A)** The bioluminescent image was obtained from four fish at 0, 6, 12, 24 h, and subsequent daily intervals until 14 days. 0 h picture was taken after 1 h immersion challenge of catfish with mutants and *Ei*WT. One of the four catfish challenged with *Ei*WT died at 12 h post-infection. **(B)** Total photon emissions obtained from the images up to 72 h post-infection. Total photon emissions were collected by IVIS Lumina XRMS In Vivo Imaging System Series III at an exposure time of 1 min.

The bioluminescent imaging was used to monitor the persistence of *Ei*Δ*evpC, Ei*Δ*hcp2*, and *Ei*Δ*evpC*Δ*hcp2* in catfish fingerlings. *Ei*WT was able to kill all catfish fingerlings in 5 days shortly after ESC clinical signs were observed (Figure 3A). Catfish fingerlings exposed to *Ei*Δ*evpC* and *Ei*Δ*evpC*Δ*hcp2* mutants survived, and clearance of mutants from the catfish fingerlings was observed. However, the immersion challenge of *Ei*Δ*hcp2* showed severe mortality of all catfish fingerlings in 8 days (Figure 3A). The bioluminescent photon counts from fingerlings showed that the number of *Ei*Δ*evpC* and *Ei*Δ*evpC*Δ*hcp2* had peaked at the highest point at 48 h (Figure 3B). On the other hand, the bioluminescence of *Ei*Δ*hcp2* was continued to increase after 48 h post-infection (Figure 3B). These findings demonstrated that *Ei*Δ*evpC* and *Ei*Δ*evpC*Δ*hcp2* were attenuated and cleared from catfish fingerlings while *Ei*Δ*hcp2* was not attenuated.

### Bacterial Killing of the Mutants in Catfish Peritoneal Macrophages

**Figure 4:**
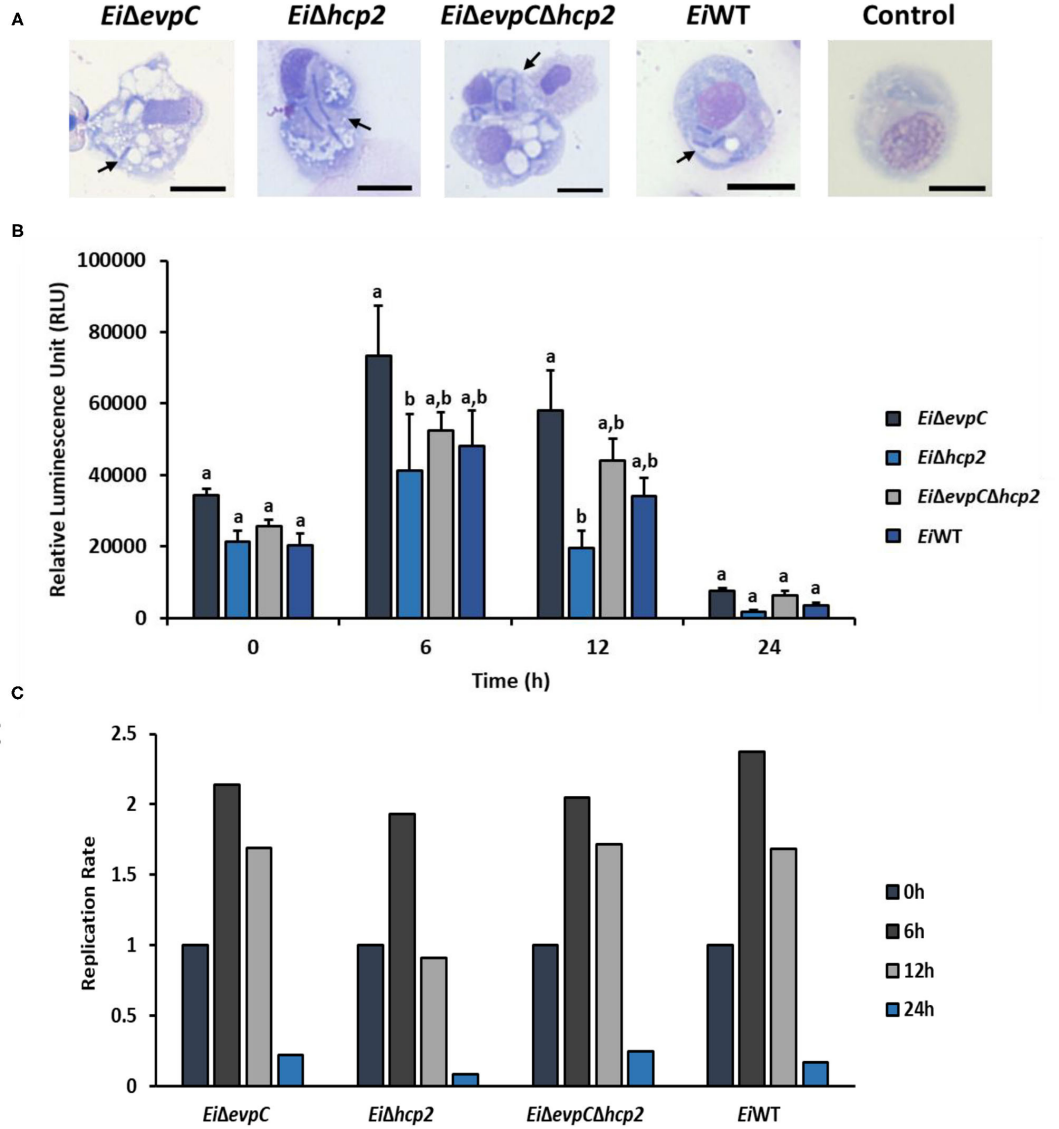
Bacterial killing assay of *Ei*Δ*evpC, Ei*Δ*hcp2, Ei*Δ*evpC*Δ*hcp2*, and *Ei*WT. **(A)** Active uptake of mutants and *Ei*WT in catfish peritoneal macrophages at 4 h post-treatment (100X). Black arrows mark engulfed bacteria in the cytoplasm and phagosomes and/or phagolysosomes of peritoneal macrophages. Scale bar = 10 μm. **(B)** The bar graph indicates mean relative luminescence unit (RLU) of four biological replicas obtained by Cytation 5 at an exposure time of 1 min. The graph represents the mean bioluminescence of each treatment ± SD. Letters above bars show the significant differences between treatments (*p* < 0.05). **(C)** Replication rate of mutants and *Ei*WT in catfish peritoneal macrophages, which is calculated from RLU data in B by dividing RLU at 6, 12, and 24 h to RLU at 0 h.

*Ei*WT and mutant strains were observed in phagosome/phagolysosome and cytoplasm of peritoneal macrophages by light microscopy (Figure 4A). The intensity of bacterial bioluminescence in catfish macrophages did not differ among the treatment groups at 0 h (Figure 4B). However, the luminescence of bacteria increased in all treatments at 6 h post-treatment. The intensity of luminescence from *Ei*Δ*hcp2* was significantly lower than that of *Ei*Δ*evpC* at this time point (Figure 4B). Interestingly, bacterial luminescence decreased in all groups at 12 h post-treatment, and the luminescence of *Ei*Δ*hcp2* was lower significantly compared to *Ei*Δ*evpC* at this time point. However, there were no significant differences in the intensity of luminescence between *Ei*Δ*hcp2* and *Ei*WT and *Ei*Δ*evpC*Δ*hcp2* at both 6 and 12 h post-treatment (Figure 4B). After 24 h, bacterial luminescence decreased in all treatment groups, and there were no significant differences between the treatments (Figure 4B). Our results suggest that *Ei*WT and Hcp mutants are capable of surviving and replicating in catfish peritoneal macrophages up to 6 h post-treatment. However, peritoneal macrophages efficiently killed *Ei*WT and Hcp mutant strains after 24 h of *in vitro* infection (Figure 4C).

### Attachment and Invasion Capabilities of the Mutants in CCO Cells

**Figure 5:**
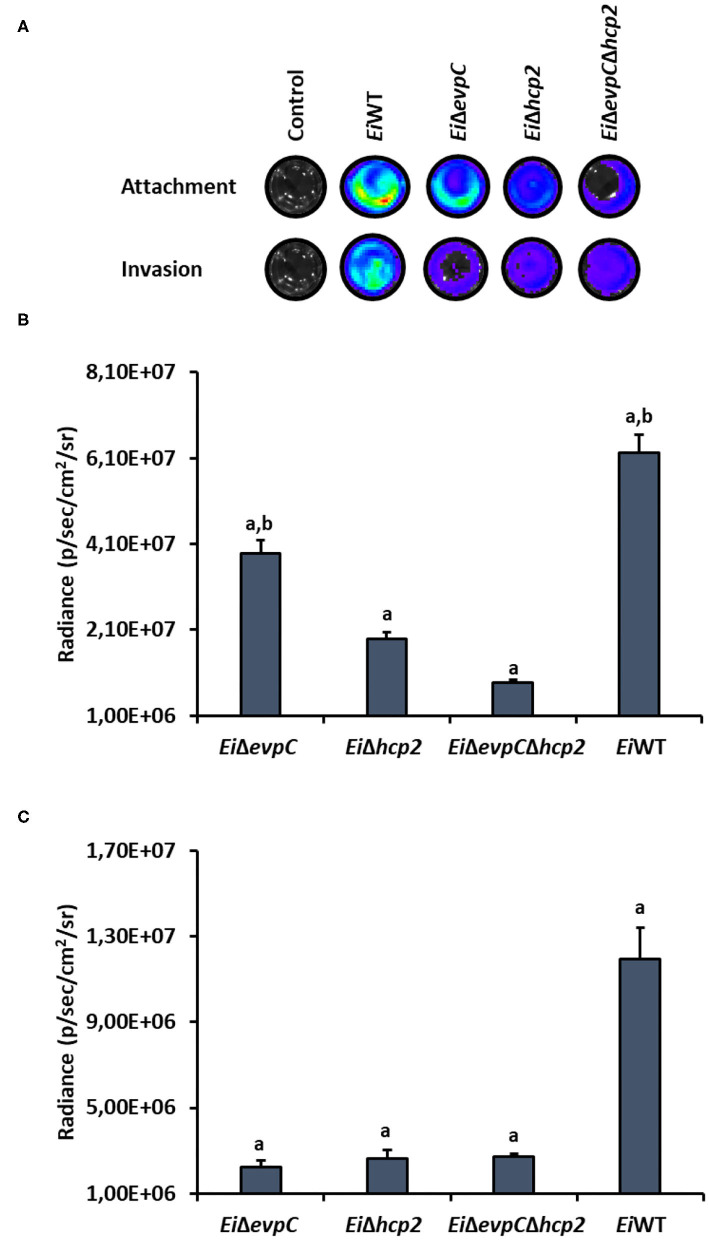
Attachment and invasion of bioluminescent *Ei*Δ*evpC, Ei*Δ*hcp2, Ei*Δ*evpC*Δ*hcp2*, and *Ei*WT in CCO cells. **(A)** Attachment and invasion of *E. ictaluri* strains in CCO cells. Total photon emissions from the CCO cells were collected by IVIS Lumina XRMS In Vivo Imaging System Series III at an exposure time of 1 min. **(B)** Attachment: The bar graph indicates the mean photon emissions from four biological replicates after 1 h post-infection. Letters show the significant differences between treatments (*p* < 0.05). **(C)** Invasion: The bar graph indicates the mean photon emissions from four biological replicates after 1 h incubation in media containing gentamycin.

CCO cell line was used to assess the attachment and invasion capabilities of *Ei*WT and mutants, *Ei*Δ*evpC, Ei*Δ*hcp2*, and *Ei*Δ*evpC*Δ*hcp2* (Figure 5A). The attachment ability of *Ei*Δ*hcp2* and *Ei*Δ*evpC*Δ*hcp2* significantly declined compared to *Ei*WT (*p* < 0.05; Figure 5B). However, no significant differences were recorded between *Ei*Δ*evpC* and *Ei*WT (*p* < 0.05). In addition to the attachment, invasion of all mutants was reduced, but there were no significant differences compared to *Ei*WT (*p* < 0.05; Figure 5C). These results indicate that Hcp mutants resulted in low attachment and invasion capabilities.

### Survival and Stress Resistance of the Mutants in BHI and MM19-P

**Figure 6:**
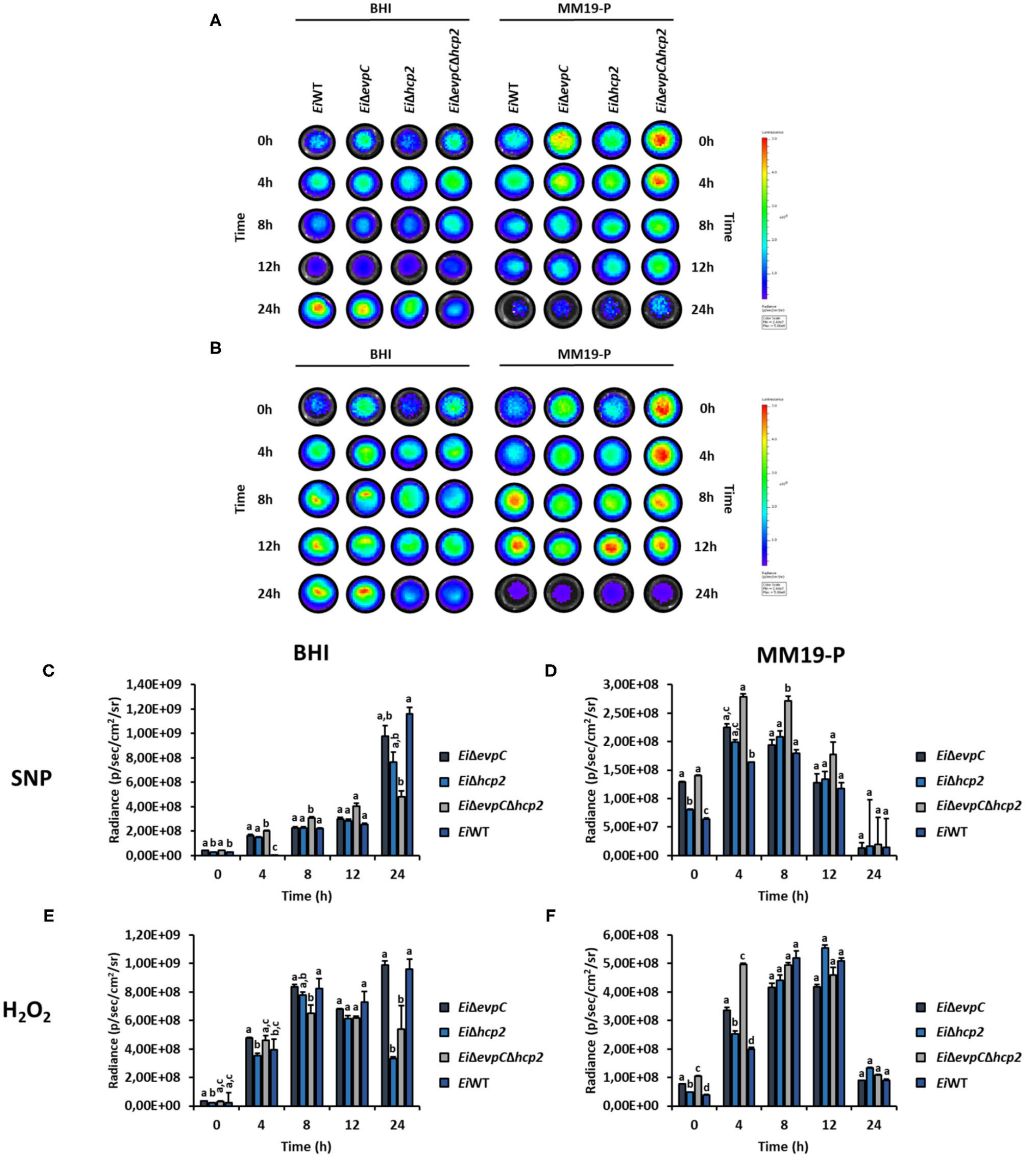
Survival and stress resistance of *Ei*Δ*evpC, Ei*Δ*hcp2, Ei*Δ*evpC*Δ*hcp2*, and *Ei*WT in BHI and MM19. Bioluminescent imaging of mutants and *Ei*WT exposed to H_2_O_2_ **(A)** and SNP **(B)** in BHI and MM19 for 24 h. The picture represents one of three biological replicates. Total photon emissions from *E. ictaluri* under stress were collected by IVIS Lumina XRMS In Vivo Imaging System Series III at an exposure time of 1 min. **(C,D)** Total photon counts obtained from bioluminescent mutants and *Ei*WT exposed to SNP in BHI and MM19 for 24 h. **(E,F)** Total photon counts obtained from bioluminescent mutants and *Ei*WT exposed to H_2_O_2_ in BHI and MM19 for 24 h. Data represent the mean of three biological replicates ± SD. Letters show the significant differences between treatments at each time point (*p* < 0.05).

The survival and resistance of the *Ei*WT and mutants to nitrite oxide and hydrogen peroxide were evaluated. The exposure of mutants and *Ei*WT to SNP and H_2_O_2_ in BHI and MM19-P showed a variation in the growth rate of bacteria (Figures 6A,B). Their resistance was increased in MM19-P compared to BHI up to 12 h (Figures 6C,E). Due to the low pH (5.5) in MM19-P, the resistance of mutants and *Ei*WT was enhanced in 0 and 4 h. The mutants and *Ei*WT strains grew exponentially up to 24 h in BHI whereas their growth was restricted in MM19-P at 24 h (Figures 6D,F). *Ei*Δ*evpC*Δ*hcp2* double mutant had more resistance to SNP and H_2_O_2_ stress in BHI and MM19-P up to 12 h. However, *Ei*Δ*evpC* and *Ei*Δ*hcp2* showed a similar growth rate in BHI and MM19-P.

### Assessment of Virulence and Efficacy of the Mutants in Catfish

**Figure 7:**
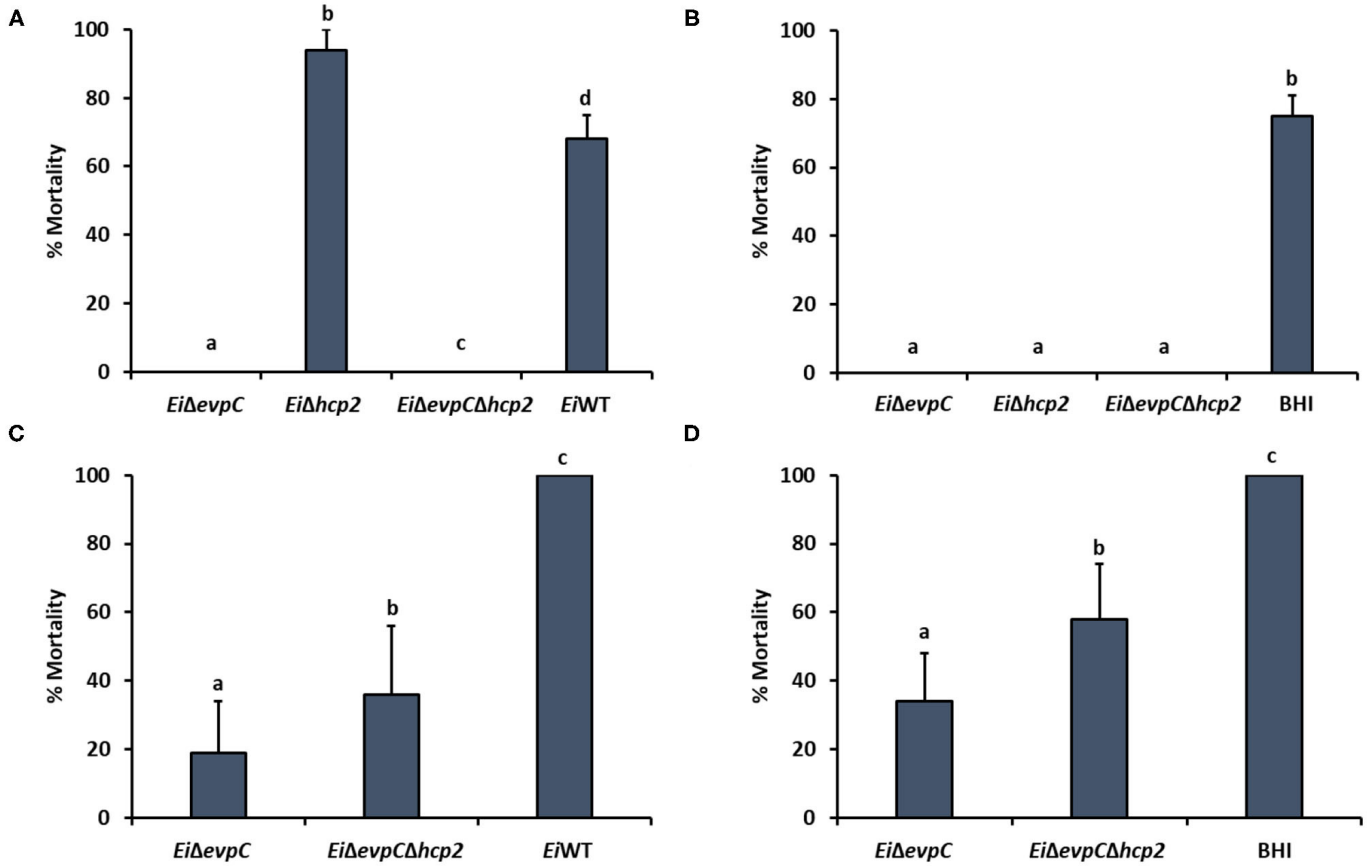
Virulence and vaccine efficacy of *Ei*Δ*evpC, Ei*Δ*hcp2, Ei*Δ*evpC*Δ*hcp2*, and *Ei*WT in catfish. Percent mortality in virulence **(A)** and efficacy **(B)** of *Ei*Δ*evpC, Ei*Δ*hcp2, Ei*Δ*evpC*Δ*hcp2* mutants, and *Ei*WT in catfish fingerlings. Fish mortalities were recorded for 21 days. Percent mortality in virulence **(C)** and efficacy **(D)** of mutants and *Ei*WT in catfish fry. Fish mortalities were recorded for 21 days.

The immersion challenge was used to evaluate mutants' virulence and efficacy in catfish fingerlings and fry (Figure 7). *Ei*Δ*evpC* (0% mortality) and *Ei*Δ*evpC*Δ*hcp2* (0% mortality) were completely attenuated in catfish fingerlings in comparison to *Ei*WT (67.53% mortality) (*p* < 0.05). Interestingly, *Ei*Δ*hcp2* caused a severe and rapid death (93.94% mortality) in catfish fingerlings (Figure 7A). The protection of *Ei*Δ*evpC* (0 % mortality) and *Ei*Δ*evpC*Δ*hcp2* (0% mortality) were significantly better than *Ei*WT (Figure 7B). In catfish fry challenge, virulence of *Ei*Δ*evpC* (18.72% mortality) and *Ei*Δ*evpC*Δ*hcp2* (35.90% mortality) decreased significantly in comparison to *Ei*WT (100% mortality) (Figure 7C; *p* < 0.05). These mutants showed protection in fry compared to the sham-vaccinated group (Figure 7D). The protection of *Ei*Δ*evpC* (33.93% mortality) was better than that of *Ei*Δ*evpC*Δ*hcp2* (58.42% mortality).

## Discussion

This research aimed to determine the potential roles of *hcp1* (*evpC*) and *hcp2* of T6SS in *E. ictaluri* virulence in catfish. To achieve this, *Ei*Δ*evpC, Ei*Δ*hcp2*, and *Ei*Δ*evpC*Δ*hcp2* mutants were constructed and persistence in catfish, survival and replication inside catfish peritoneal macrophages, attachment and invasion capabilities in catfish epithelial cells, adaptation and survival to stress factors, and virulence and efficacy in catfish were investigated (Table 3).

**Table 3:** Mutants of Hcp family genes of T6SS in *E. ictaluri*.

			**BKA**		**CCO**		**Stress Assays**				**Vaccination**			
	**Serum**	**BLI**	**Uptake**	**Survival**	**Attachment**	**Invasion**	**BHI**		**MM19-P**		**Virulence**		**Efficacy**	
							**SNP**	**H_**2**_O_**2**_**	**SNP**	**H_**2**_O_**2**_**	**Fingerling**	**Fry**	**Fingerling**	**Fry**
*Ei*Δ*evpC*	–	14 d	–	+	–	–	–	–	–	–	+	+	+	+
*Ei*Δ*hcp2*	–	14 d	–	+	+	–	–	–	–	–	+	+	+	+
*Ei*Δ*evpC*Δ*hcp2*	–	8 d	–	–	+	–	+	+	+	–	+	+	+	+

*Serum, Catfish normal serum treatment at 4 h; BLI, Real-time bioluminescent imaging (persistency of bacteria in 2-weeks); BKA, Bacterial killing assay (peritoneal macrophages), uptake at 0 h and survival at 6 h; CCO, Channel catfish ovary cells (epithelial cells); Stress Assays, Treated with stress factors at 8 h; BHI, Brain-heart infusion broth (rich medium); MM19-P, Low-phosphate minimal medium broth at pH 5.5 (minimal medium); SNP, Sodium nitroprusside (nitrosative stress); H_2_O_2_, Hydrogen peroxide (oxidative stress); Virulence, Catfish immersion challenged with mutants and EiWT for 21 days; Efficacy, Catfish immersion re-challenged with EiWT for 21 days; Fingerling, Six-month-old catfish; Fry, Two-week-old catfish. (+) shows difference and (–) shows no difference compared to EiWT (p < 0.05)*.

Almost all strains of *E. ictaluri* show beta-type homolysis and hemolytic zone is narrow (36). Hemolytic activity can vary between strains and there is no clear correlation between hemolytic activity and virulence (37, 38). Our study indicated that hemolytic activities of *Ei*Δ*evpC, Ei*Δ*hcp2*, and *Ei*Δ*evpC*Δ*hcp2* were similar to *Ei*WT, and deletion of *evpC* and *hcp2* genes did not have any effect on *E. ictaluri* hemolytic activity. Hemolysin co-regulated family proteins (Hcp) are involved in adhesion and invasion, intracellular survival of bacteria, bacterial cytotoxicity, and virulence (16).

*Edwardsiella ictaluri* can evade the complement system in catfish serum and establish a systemic infection. *Edwardsiella ictaluri* can differentially regulate its proteins in catfish serum (39). Our study revealed that *Ei*Δ*evpC, Ei*Δ*hcp2*, and *Ei*Δ*evpC*Δ*hcp2* were resistant to complement killing in catfish blood. Mutation in *evpC, hcp2*, and *evpC*-*hcp2* did not affect *E. ictaluri*'s resistance to complement killing, which indicates that Hcp family proteins of T6SS are not essential for *E. ictaluri* to survive in catfish serum.

The real-time bioluminescent imaging is a quantification method that allows detection *E. ictaluri* infection and persistence of mutants in catfish (40, 41). The bioluminescence from *Ei*Δ*evpC* (7.5 × 10^4^ photons^−1^ cm^−2^ steradian^−1^) and *Ei*Δ*evpC*Δ*hcp2* (3.6 × 10^4^ photons^−1^ cm^−2^ steradian^−1^) was reached the peak at 48 h post-infection, after which bacterial clearance from catfish was observed. However, bioluminescence from *Ei*Δ*hcp2* (1.4 × 10^5^ photons^−1^ cm^−2^ steradian^−1^) and *Ei*WT (1.6 × 10^5^ photons^−1^ cm^−2^ steradian^−1^) was gradually increased, even after 48 h post-infection, until the fish dies. Our result showed that *Ei*Δ*evpC* and *Ei*Δ*evpC*Δ*hcp2* had no mortalities for 14 days, although the bioluminescence quantity of *Ei*Δ*evpC* and *Ei*Δ*evpC*Δ*hcp2* started to increase earlier than *Ei*Δ*hcp2* and *Ei*WT. Our bioluminescent imaging data were corroborated with our virulence and efficacy study showing that persistence and replication of *Ei*Δ*evpC* and *Ei*Δ*evpC*Δ*hcp2* in catfish up to 48 h post-infection and decrease afterward may stimulate catfish an immune response, hence the survival of catfish.

Hcp family proteins are secreted inside host macrophages and required for intracellular survival in host macrophages (42, 43). Lack of a functional *hcp* reduced survival of *Burkholderia pseudomallei* in macrophages (44, 45). In *E. tarda*, the deletion of *evpC* caused a lower replication rate in gourami phagocytes (46). In this study, we found that the mutation in *hcp2* displayed a lower replication rate for the intracellular growth of *E. ictaluri* inside the catfish peritoneal macrophages. The numbers of macrophages with intracellular *Ei*Δ*evpC, Ei*Δ*hcp2, Ei*Δ*evpC*Δ*hcp2*, and *Ei*WT bacteria were similar at 0 and 24 h post-infection. At 6 and 12 h post-infection, the number of cells with intracellular *Ei*Δ*evpC* and *Ei*WT were similar, but intracellular replication of *Ei*Δ*hcp2* in macrophages was significantly impaired. This result suggests that *hcp2* may be necessary for *E. ictaluri* replication within catfish macrophages. This may depend on lack of *hcp2* or a *hcp2*-dependent effector protein, which warrant further investigation.

Hcp family proteins are involved in adherence and invasion of the host epithelial tissues. Disruption in *hcp* genes could cause different results in Hcp-mediated cell adhesion and invasion activity of pathogenic bacteria. Hcp mutants displayed reduced adhesion and invasion of epithelial cells in *Campylobacter jejuni, E. coli*, and *Vibrio parahaemolyticus* (47–49). However, the deletion of *hcp* caused increased adhesion and invasion of MODE-K cell line in *Helicobacter hepaticus* (50). We demonstrated that *evpC* and *hcp2* mutants' adhesion capabilities were dissimilar while their invasion capabilities were similar in CCO cells. Mutation in *evpC* did not decrease adherence to CCO cells while a mutation in *hcp2* and both in *evpC* and *hcp2* did. This suggests that *hcp2* is required for epithelial cell attachment of *E. ictaluri* whereas both *evpC* and *hcp2* are not essential for epithelial cell invasion of *E. ictaluri*.

T6SS facilitates the uptake of important metals under stress conditions by releasing proteinaceous metallophores into the host environment (51). The role of T6SS in manganese scavenging under oxidative stress has been revealed in *Burkholderia thailandensis* (52). Intracellular compartmentalization of *Salmonella typhimurium* inside macrophages initiates stress conditions, including nitrosative and oxidative stress, to suppress the replication of bacteria (53). T6SS effectors are involved in bacterial survival in oxidative stress (54, 55). In *E. ictaluri* and *E. piscicida*, the T6SS effector EvpP enhanced resistance to oxidative stress (56, 57). To investigate the role of Hcp family proteins of T6SS in *E. ictaluri* stress resistance, we applied nitrosative and oxidative stress with SNP and H_2_O_2_ in BHI and in MM19-P to imitate stressful phagosome conditions. Our results indicated that mutants and *Ei*WT were able to grow in nutrient rich media, but SNP and H_2_O_2_ stress reduced survival of *Ei*Δ*hcp2* and *Ei*Δ*evpC*Δ*hcp2* at 24 h. It seems that *hcp2* is more critical for *E. ictaluri* to cope with SNP and H_2_O_2_ stress in presence of nutrients. In nutrient restricted media, mutants and *Ei*WT were able to grow up to 4 h, but SNP and H_2_O_2_ stress suppressed *Ei*WT growth more than mutants, which may indicate both *evpC* and *hcp2* are not critical in phagosome conditions.

*In vivo* and *in vitro* infection models indicated that Hcp family proteins were associated with bacterial virulence and host colonization. In *Aeromonas hydrophila, E. coli*, and *B. pseudomallei, hcp* is required for virulence because *hcp* mutants were less virulent than wild-type (58, 59). Additionally, *evpC* was essential for the virulence of *E. tarda* (18). Here, we showed that *evpC* contributed to the pathogenicity of *E. ictaluri* in catfish. Vaccination of catfish fingerlings with *Ei*Δ*evpC* and *Ei*Δ*evpC*Δ*hcp2* provided complete protection against ESC in catfish fingerlings. However, *Ei*Δ*hcp2* showed a hypervirulent phenotype causing higher mortality with severe symptoms in catfish fingerlings and was not tested in catfish fry. The mortality rates of *Ei*Δ*evpC* and *Ei*Δ*evpC*Δ*hcp2* in catfish fry immersion challenge indicated that *Ei*Δ*evpC* showed significantly less mortality and better protection compared to *Ei*Δ*evpC*Δ*hcp2*.

**Figure 8:**
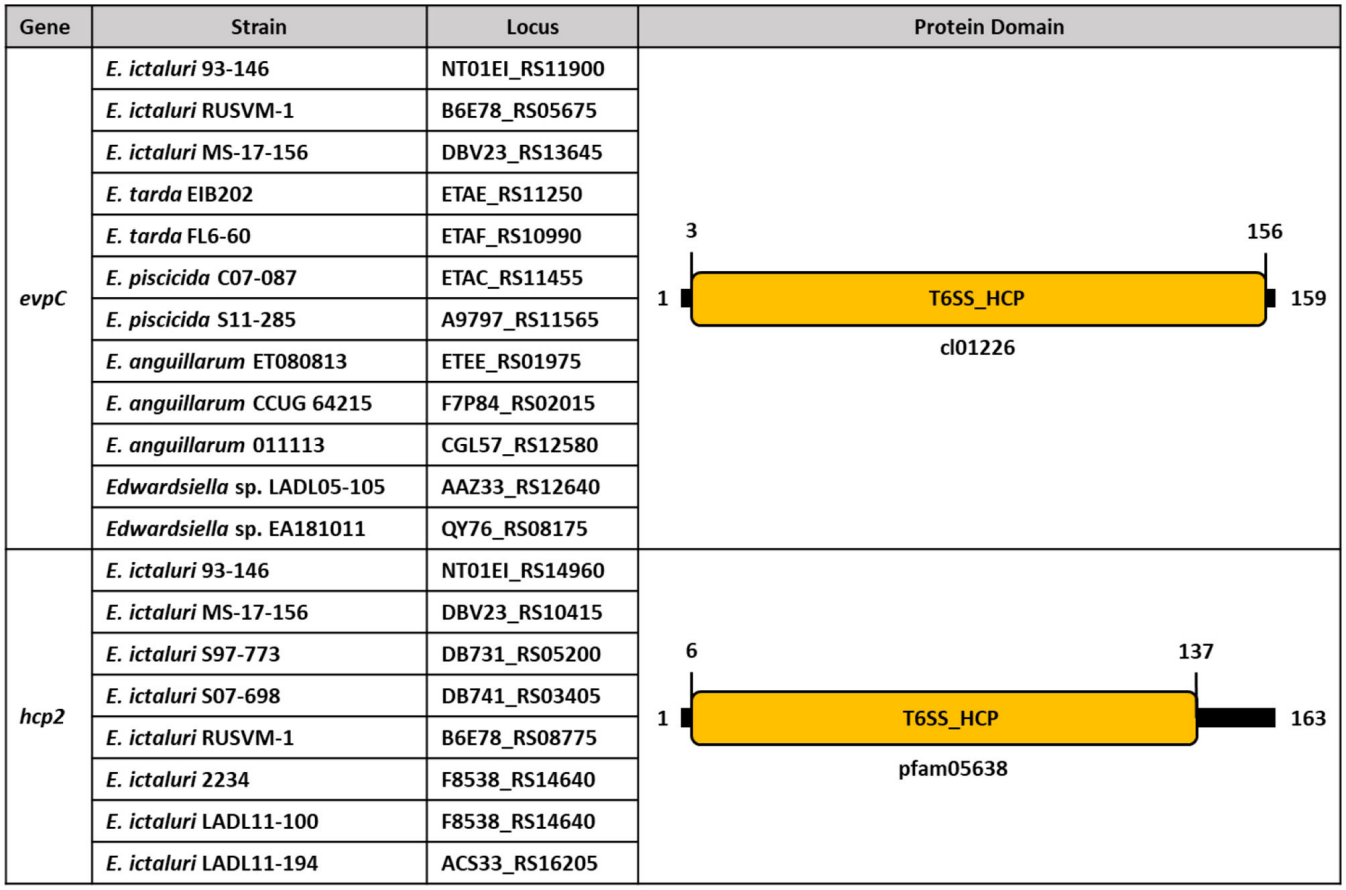
Conserved protein domains and families of *evpC* and *hcp2* in *Edwardsiella species*. Schematic representation of putative *hcp1* (*evpC*) and *hcp2* of Hcp family proteins in *E. ictaluri*. Protein domains and families were identified by using NCBI CDD and colored. Protein length is represented by black bars, and numbers indicate protein and domain sizes. Protein family and domain names are inside and below the rectangles.

Although *evpC* is located in the T6SS operon of *E. ictaluri, hcp2* is founded ~67 kilobases (kb) away from *evpC* (Figure 8). It is possible that putative *hcp2* might be an effector protein in *E. ictaluri*. It is worth noting that the protein sequence alignment of *evpC* and *hcp2* had no significant match (data not shown). Thus, these two proteins classified in the Hcp protein family may have a different role in *E. ictaluri*.

In conclusion, the two Hcp family proteins found in the *E. ictaluri* genome seems to have diverse roles in *E. ictaluri* pathogenesis. *hcp2* is important in adherence to epithelial cells and replication within macrophages. However, *evpC* plays a crucial role in *E. ictaluri* virulence in catfish. Therefore, secretion of potential *evpC* and *hcp2* dependent effector proteins via T6SS need more investigation.

## Data Availability Statement

The original contributions presented in the study are included in the article/supplementary material, further inquiries can be directed to the corresponding author/s.

## Ethics Statement

The animal study was reviewed and approved by Institutional Animal Care and Use Committee at Mississippi State University.

## Author Contributions

SK, AOK, LP, and AK conceived and designed the experiments. SK, HA, and AOK performed the experiments. SK wrote the manuscript. All authors reviewed and approved the final version of the manuscript.

## Conflict of Interest

The authors declare that the research was conducted in the absence of any commercial or financial relationships that could be construed as a potential conflict of interest.
